# Supplementary material for: Supermagnetic Human Serum Albumin (HSA) Nanoparticles and PLGA-Based Doxorubicin Nanoformulation: A Duet for Selective Nanotherapy
Source: Int J Mol Sci. 2022 Dec 30;24(1):627. doi: 10.3390/ijms24010627 (PMC9820361; doi:10.3390/ijms24010627)
Supplement: Supplementary file 1 [file ijms-24-00627-s001.zip › Supplementary.pdf]

Supplementary materials to the article:

Supermagnetic Human Serum Albumin (HSA) Nanoparticles and PLGA-Based Doxorubicin Nanoformulation: A Duet for Selective Nanotherapy

Julia Malinovskaya [1], Rawan Salami [2,3], Marat Valikhov [4,5], Veronika Vadekhina [4], Aleksey Semyonkin [1], Alevtina Semkina [4,5], Maxim Abakumov [5], Yifat Harel [2,3], Esthy Levy [2,3], Tzuriel Levin [2,3], Rachel Persky [2], Vladimir Chekhonin [4], Jean-Paul Lellouche [2,3], Pavel Melnikov [4,†] and Svetlana Gelperina [1,*,†]

[1] Drug Delivery Systems Laboratory, D. Mendeleev University of Chemical Technology of Russia, Miusskaya pl. 9, 125047 Moscow, Russia
[2] Department of Chemistry, Faculty of Exact Sciences, Bar-Ilan University, Ramat-Gan 5290002, Israel
[3] Institute of Nanotechnology and Advanced Materials, Department of Chemistry, Faculty of Exact Sciences, Bar-Ilan University, 5290002 Ramat Gan, Israel
[4] Department of Neurobiology, V. Serbsky Federal Medical Research Centre of Psychiatry and Narcology of the Ministry of Health of the Russian Federation, Kropotkinskiy per. 23, 119034 Moscow, Russia
[5] Department of Medical Nanobiotechnology, Pirogov Russian National Research Medical University, Ostrovityanova ul 1, 117997 Moscow, Russia
* Correspondence: svetlana.gelperina@gmail.com
† These authors contributed equally to this work.

Table S1. Optical properties of fluorescently labeled PLGA and hMNP nanoparticles

Nanoparticle type	Dye content in NPs, µg/mg	Molar dye concentration, mol/mg NP	Extinction coefficient, $\ell \cdot cm^{-1} \cdot mol^{-1}$	Quantum yield	Brightness per 1 mg of NP material, $\ell \cdot cm^{-1}/mg$
hMNP-FITC	7.290	1.9E-08	74623	28.33	4.0E−5
hMNP-Cy3	10.310	1.3E-08	89660	28.00	3.3E−5
PLGA-Cy5	0.716	1.1E-09	74086	42.52	3.4E−5
PLGA-Cy3	0.694	1.17E-09	52159	73.34	4.2E−5

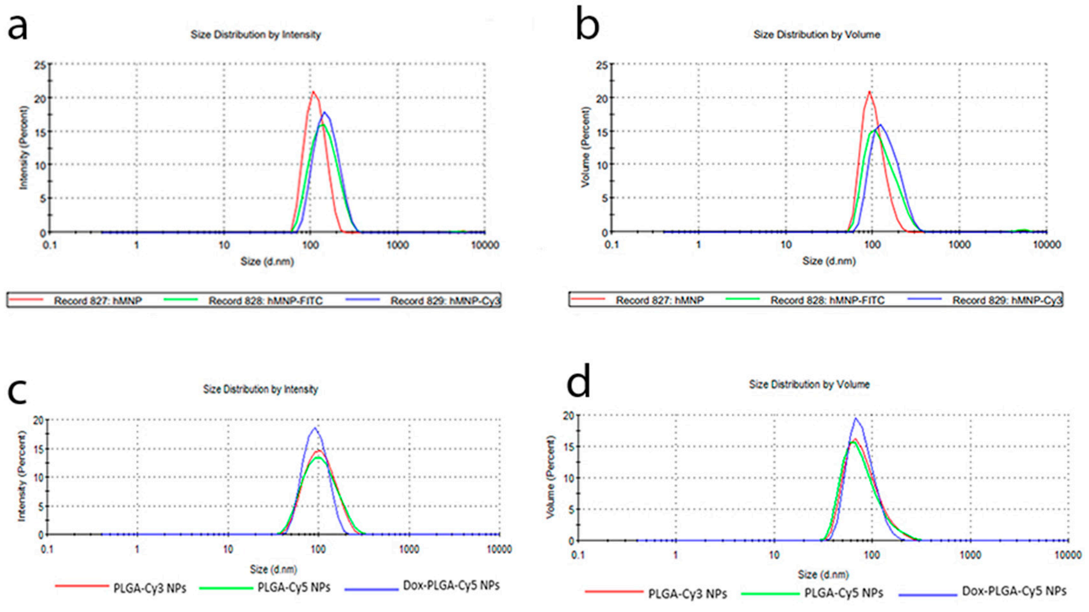

Figure S1. Size distribution (DLS) by intensity (a,c) and volume (b,d) of hMNP, hMNP-FITC and hMNP-Cy3 (red, green and blue curves, respectively) (a,b); and of PLGA-Cy3, PLGA-Cy5, Dox-PLGA-Cy5 (red, green and blue curves, respectively) (c,d). Each curve represents the mean of 4 measurements.

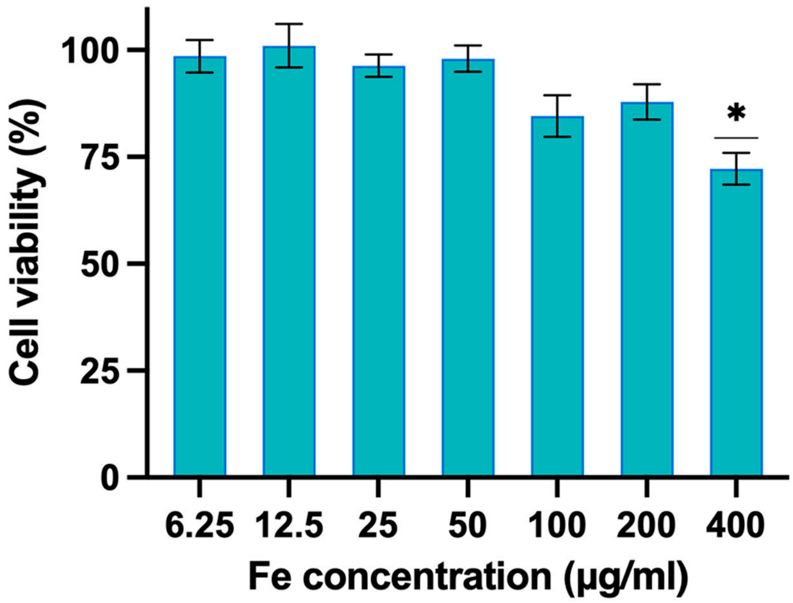

Figure S2. Evaluation of 4T1 cells viability after 24h of incubation with various concentrations of hMNP by MTS assay. Results are shown as means ± SD, *p < 0.05 (one-way ANOVA).

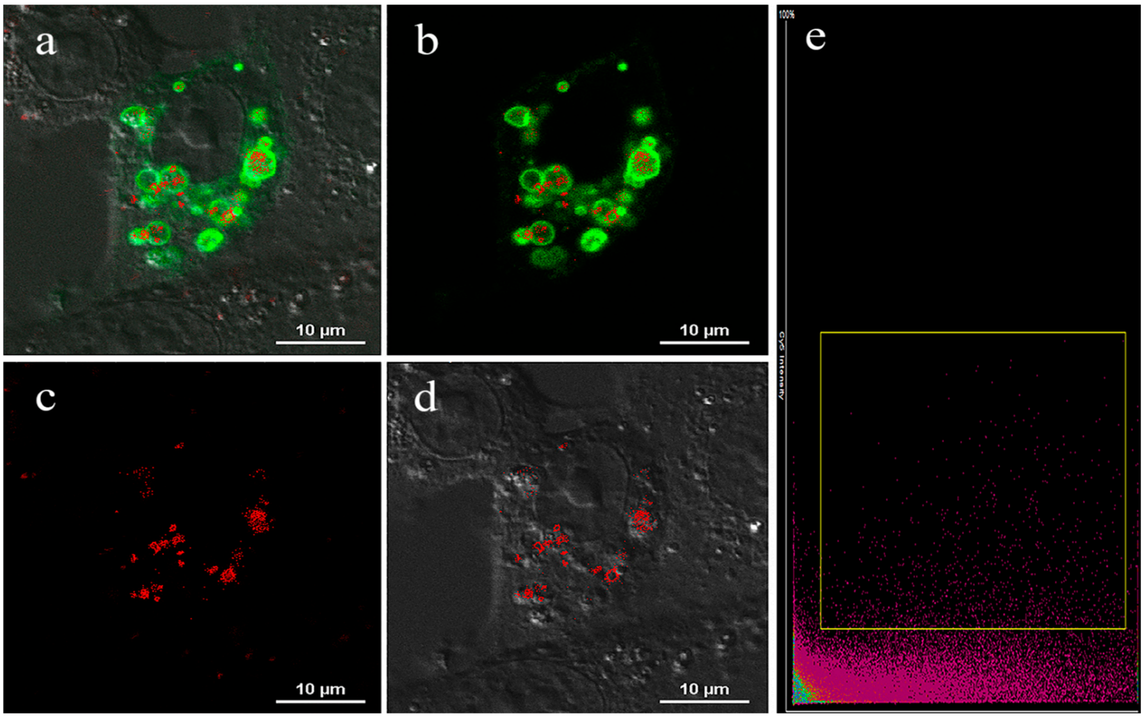

Figure S3. Evaluation of PLGA-Cy5 NP colocalization with lysosomes upon 30-min incubation with 4T1 cells by CSLM: (a) merged image; (b) Lysosomes – CellLight^{TM} Lysosomes GFP, BacMam 2.0 (green channel); (c) PLGA-Cy5 NP (red channel); (d) cell

contours – differential interference contrast (DIC), applied synthetic colocalization channel is depicted in red; (e) Scatterplot. Scale bar – 10 µm.

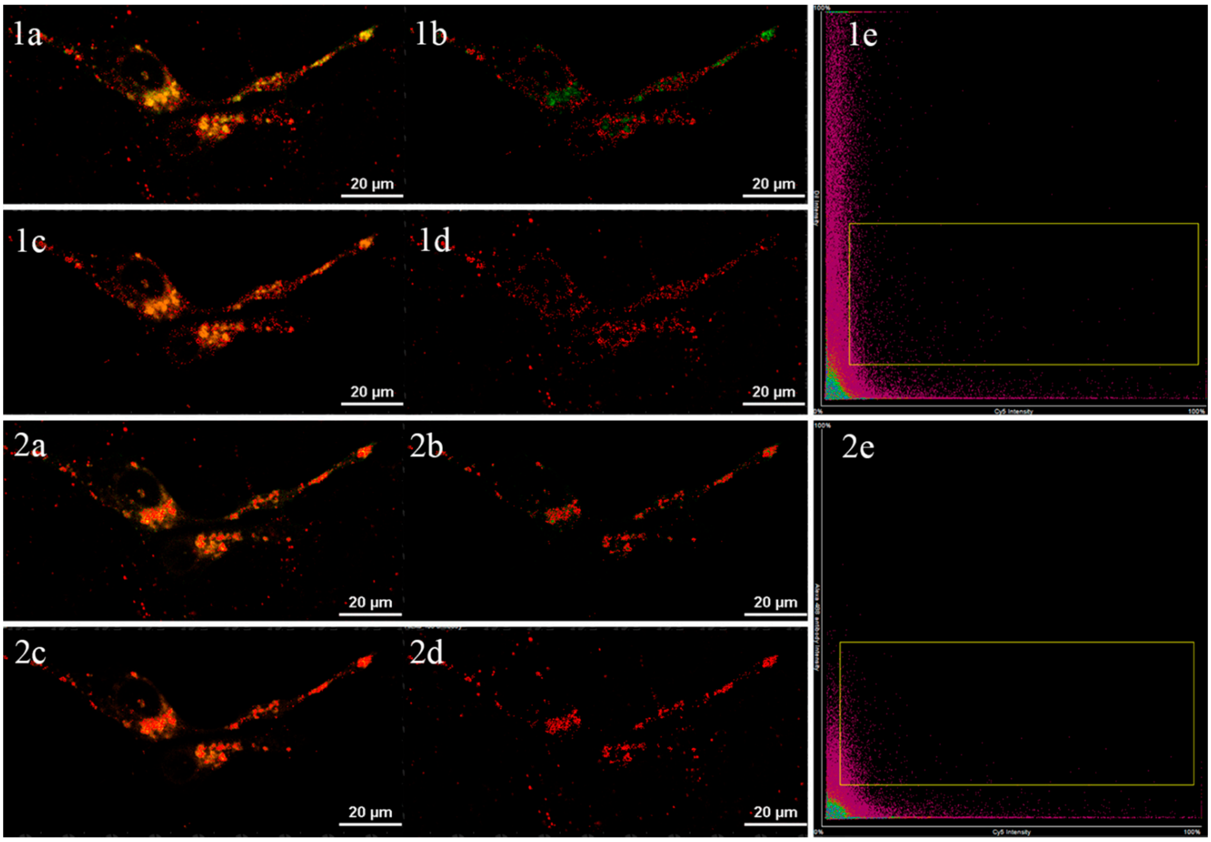

Figure S4. Evaluation of PLGA-Cy5 NP intracellular distribution upon 30-min incubation with 4T1 cells: 1 – Evaluation of PLGA-Cy5 NP colocalization with late endosomes. 2 – Evaluation of PLGA-Cy5 NP colocalization with lysosomes. (a) merged image; (b) Lysosomes – CellLight^{TM} Lysosomes GFP, BacMam 2.0 (green channel); (c) Endosomes – CellLight^{TM} Late endosomes-RFP (BacMam 2.0) (orange channel); (d) PLGA-Cy5 NP (red channel) (e) Scatterplot. Applied synthetic colocalization channel is depicted in red (a-d). Scale bar – 20 µm.

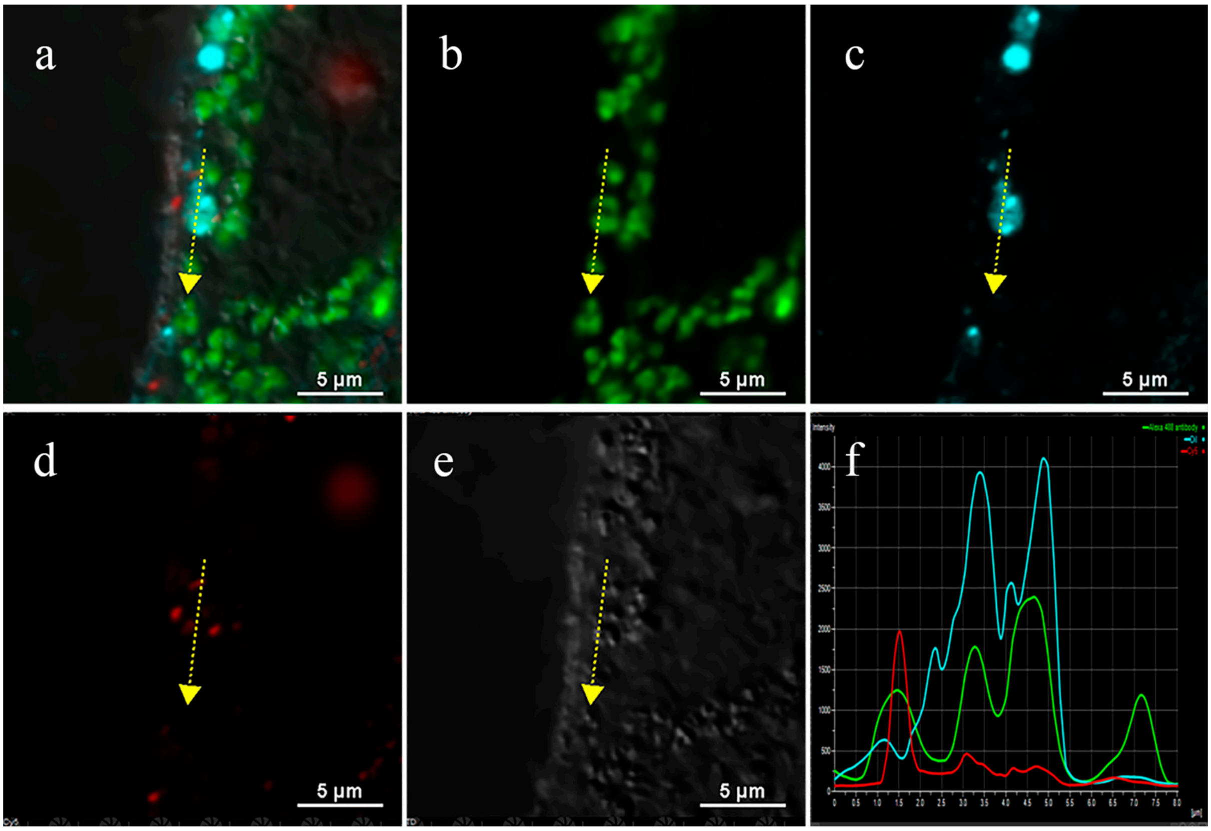

Figure S5. Investigation of PLGA-Cy5 NP and hMNP-Cy3 intracellular localization in 4T1 cells (20 min of incubation). (a) merged image; (b) Lysosomes (Lysotracker Green DND26); (c) hMNP-Cy3 (cyan channel); (d) PLGA-Cy5 NP (red channel); (e) Cell contours – differential interference contrast (DIC); (f) Fluorescence intensity profile along the vector (yellow arrow) plotted on the image. X-axis – µm, Y-axis – fluorescence intensity (standard units). Profile color corresponds to that of the channel. The overlap of green (488 nm) and red (560 nm) channels demonstrates the hMNP colocalization with lysosomes. The red peak shows low fluorescence intensity of PLGA-Cy5 NPs at that stage of incubation. Scale bar – 5 µm.

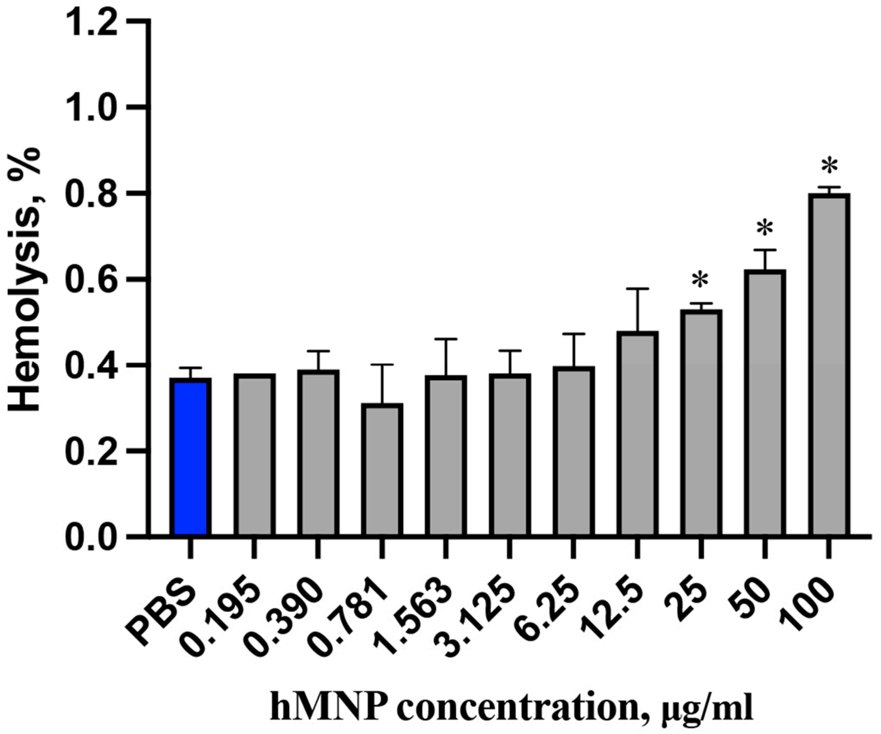

Figure S6. Percent of hemolysis after incubation of blood samples with the hMNP at different concentrations. All tested hMNP concentrations were found to be non-toxic within 2 h of incubation with erythrocytes: the percentage of hemolysis was below 1% for all samples. The data are shown as mean value ± SD (one-way ANOVA followed by Dunnett's multiple comparison test); *$p \leq 0.05$ compared with control (PBS); n=5. Positive control – cells after incubation with TritonX-100; negative control – cells after incubation with PBS solution.

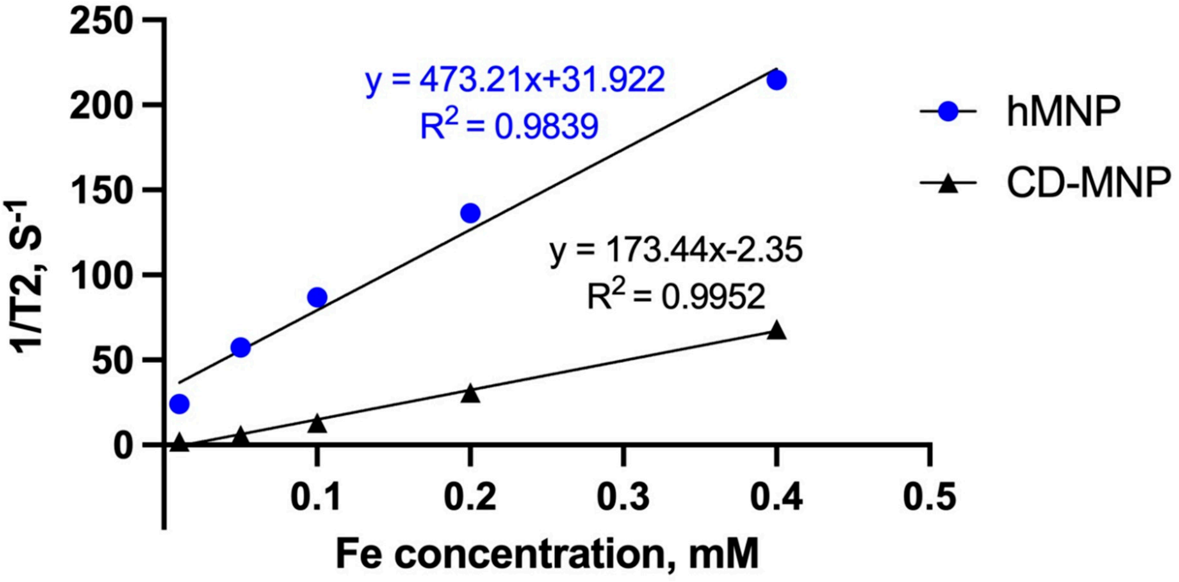

Figure S7. T2 relaxivity curves of hMNP and Absolute Mag™ Carboxyl Dextran Magnetic nanoparticles (CD-MNP).

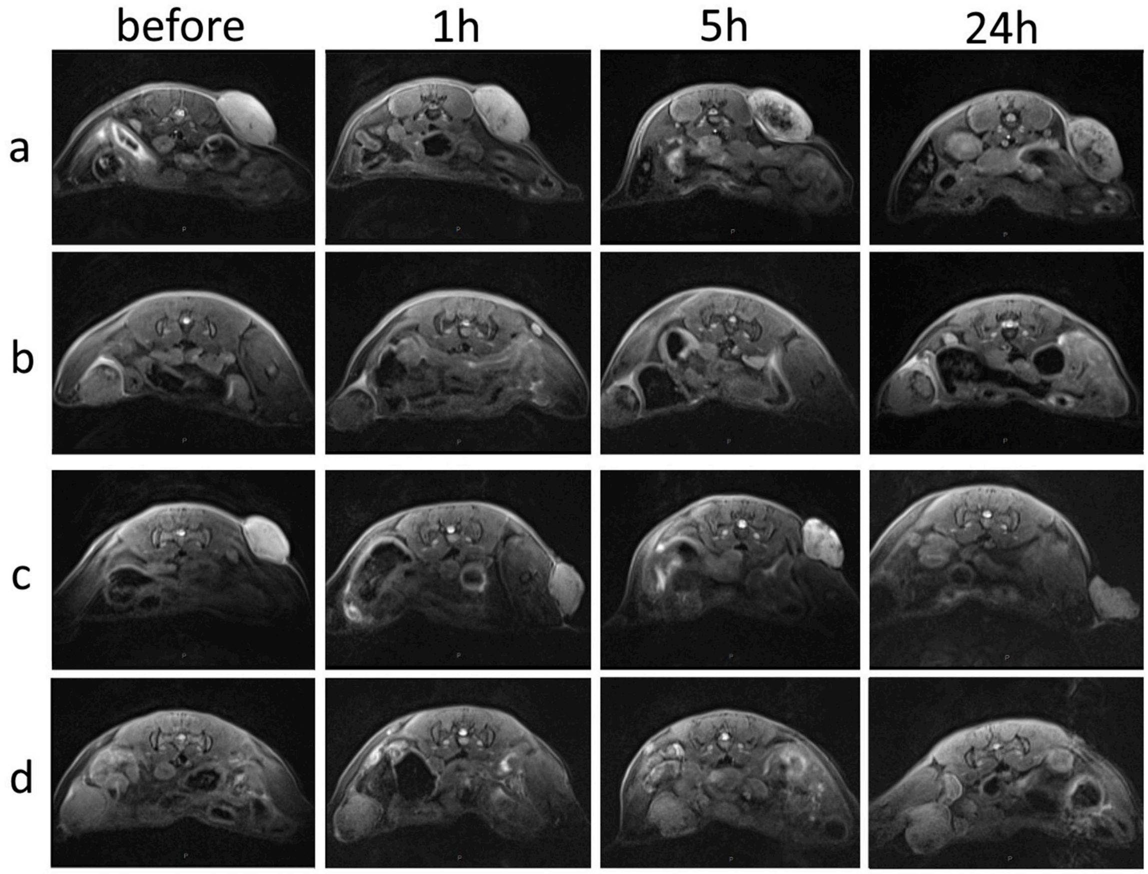

Figure S8. Representative T2-weighted images of mice with subcutaneously (a,c) and orthotopically (b,d) implanted 4T1 tumor before and 1 h, 5 h, and 14 h after administration of hMNP (a,b) and Absolute Mag™ Carboxyl Dextran Magnetic nanoparticles (c,d) (3.5mg/kg as Fe). Negatively contrasted tissues indicate hMNP accumulation. Transverse projections are demonstrated.

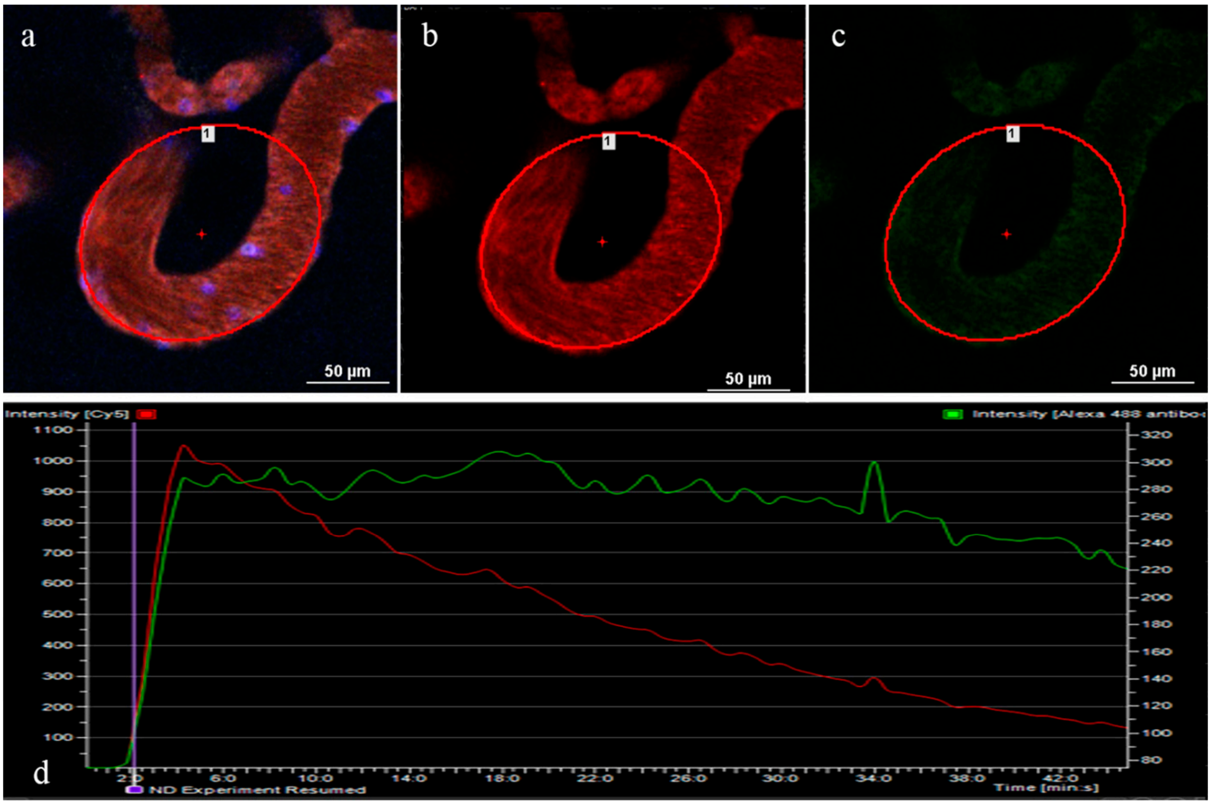

Figure S9. Blood clearance of PLGA-Cy5 NP and hMNP-FITC determined by average fluorescence intensity. The red ovals depict the ROI for evaluation of the nanoparticle fluorescence intensity in the vessel lumen. (a) Merged; (b) PLGA-Cy5 (red channel); (c) hMNP-FITC (green channel); (d) NP intravascular fluorescence intensity within 40 min. Y-axis – fluorescence intensity of PLGA-Cy5 NP (red) and hMNP-FITC (green) in intravascular ROI; X-axis – scanning period (40 min).

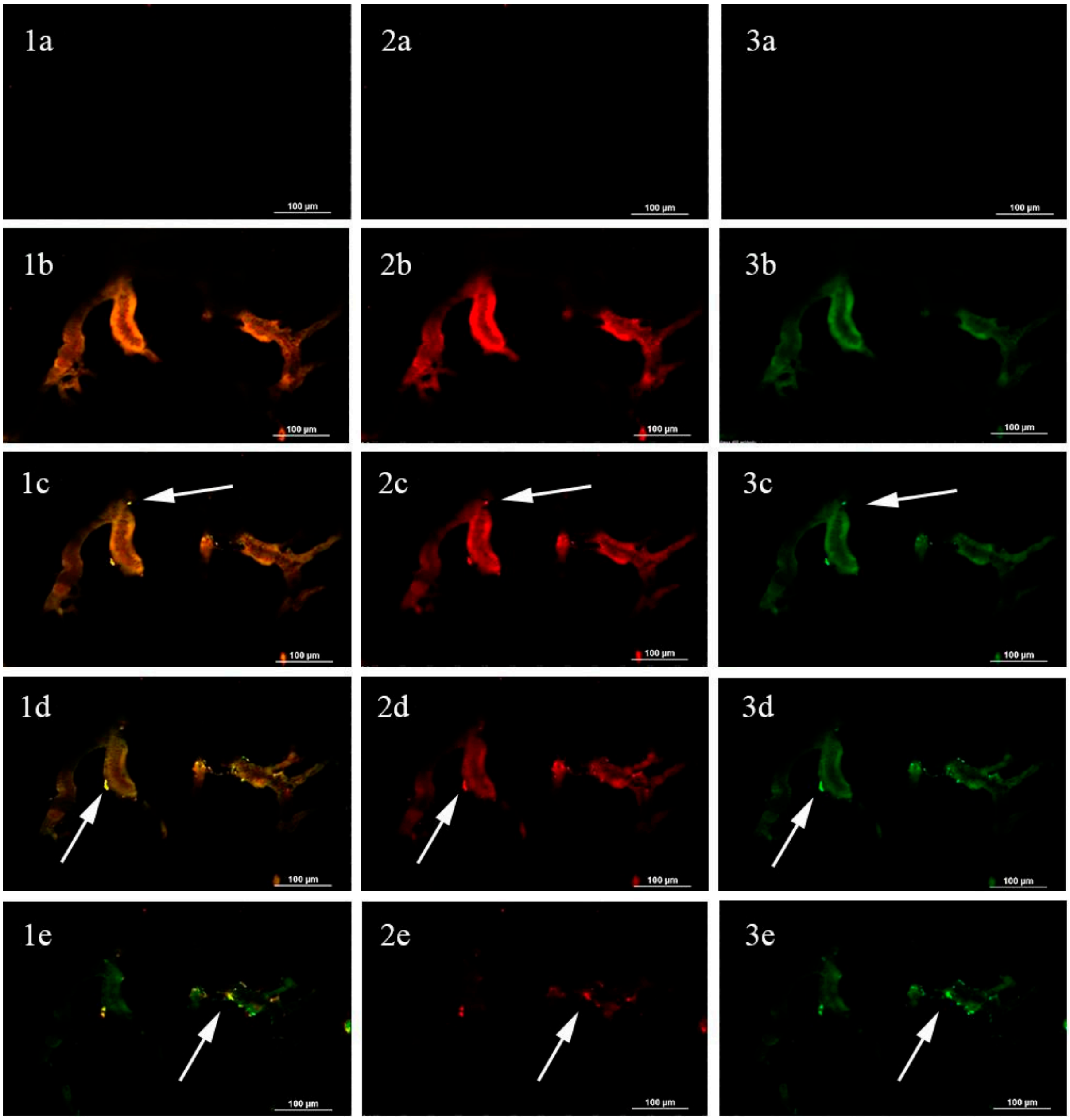

Figure S10. Evaluation of PLGA-Cy5 NP (red channel) and hMNP-FITC (green channel) distribution in the peritumoral vasculature in 4T1tumor-bearing mice upon simultaneous administration (time period 0 – 45 min). (a) 0 min; (b) 1 min; (c) 10 min; (d) 15 min; (e) 45 min. The white arrows indicate microleakage areas. (1) merged; (2) PLGA-Cy5 NP; (3) hMNP. Scale bar – 100 µm.

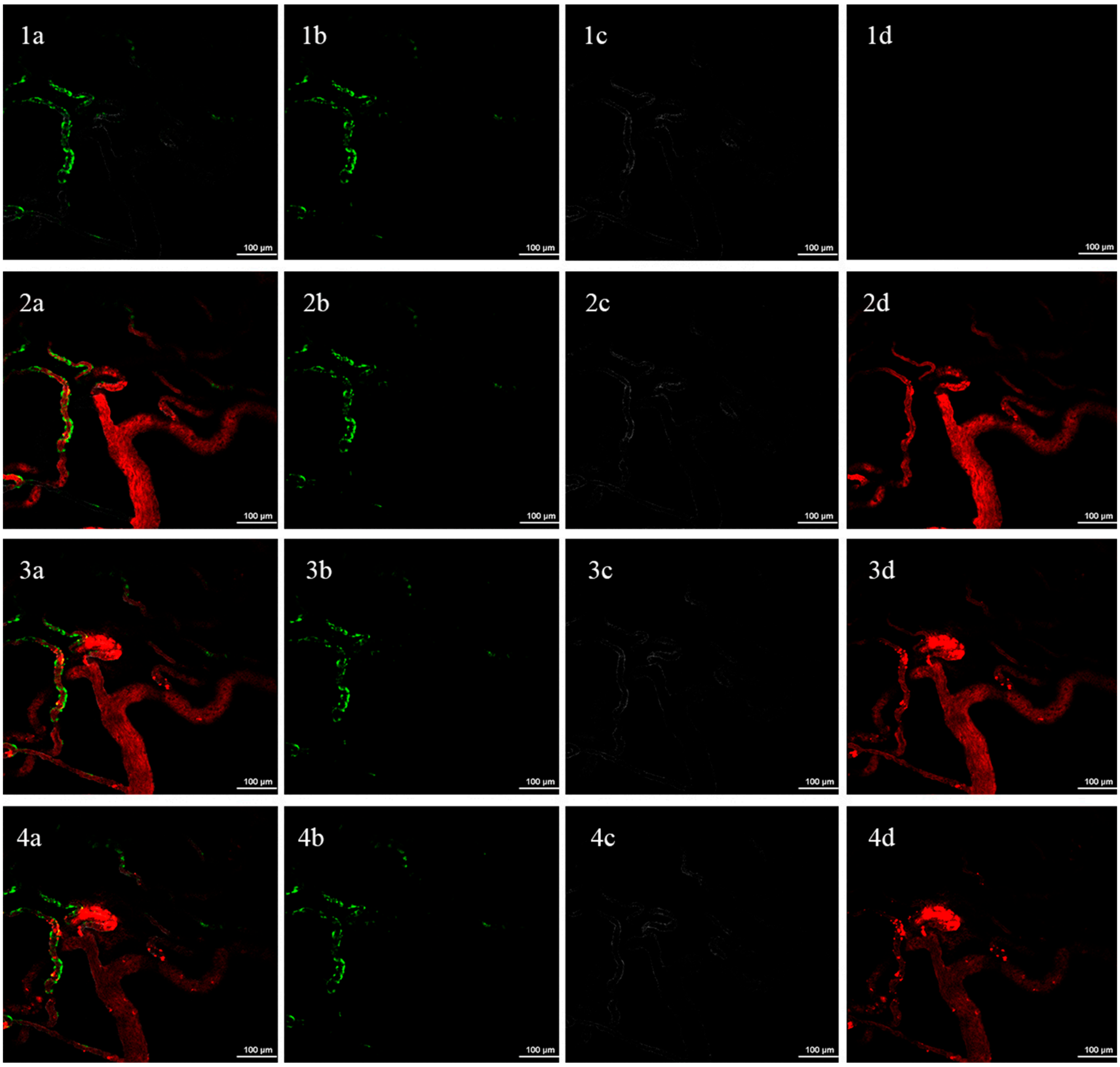

Figure S11. Evaluation of PLGA-Cy5 NP (red channel) and hMNP-FITC (green channel) distribution in the peritumoral vasculature in 4T1tumor-bearing mice (3h/1h administration scheme). The PLGA-Cy5 NP extravasation through the macroleakage and their localization compared to the hMNP accumulation is demonstrated: (a) merged; (b) hMNP-FITC; (c) CD31-PE (endothelial marker); (d) PLGA-Cy5 NP: (1) 0 min; (2) 10 min; (3) 35 min; (4) 45 min. Scale bar – 100 µm.

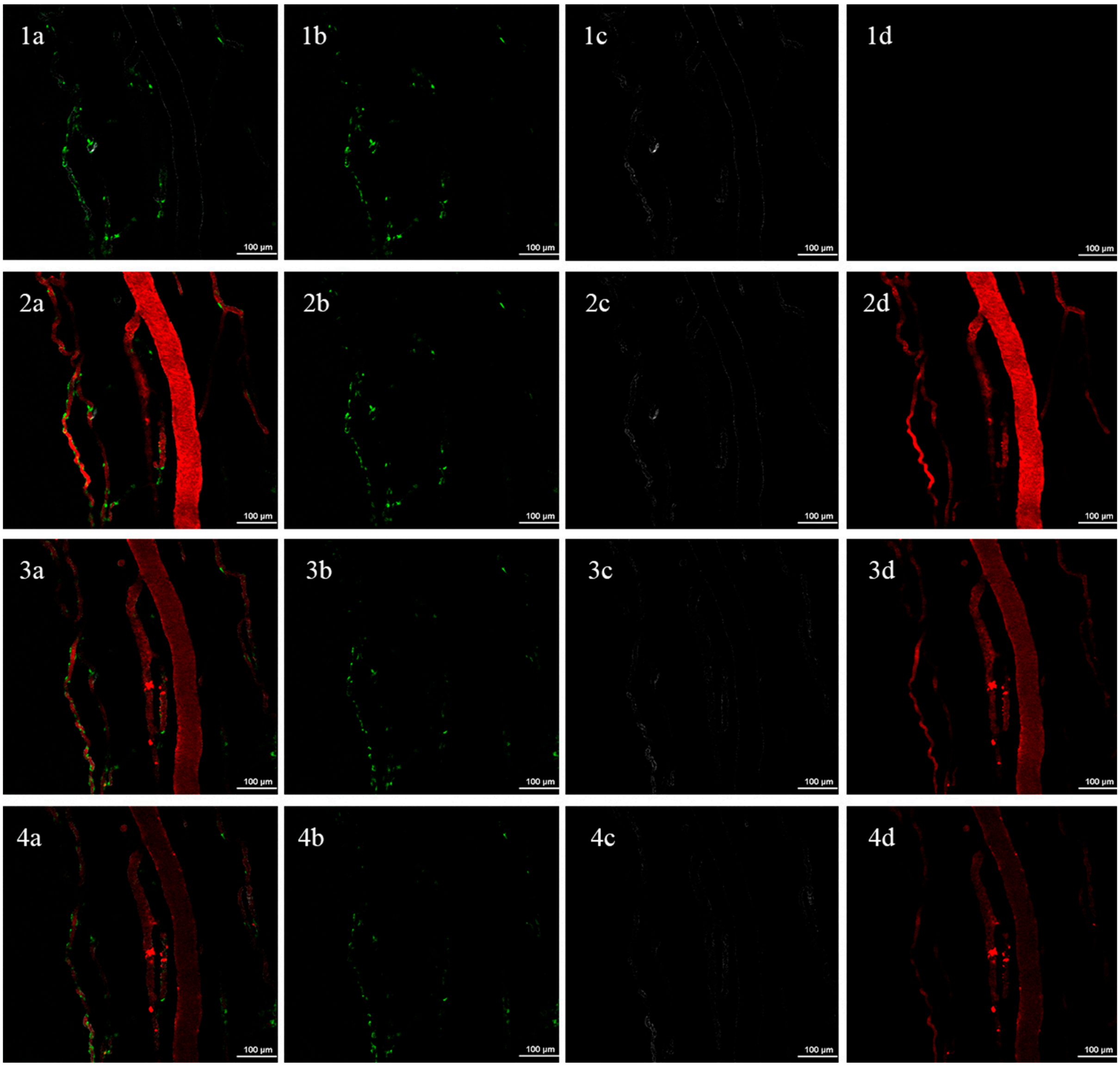

Figure S12. Evaluation of PLGA-Cy5 NP (red channel) and hMNP-FITC (green channel) distribution in the peritumoral vasculature in 4T1tumor-bearing mice (3h/1h administration scheme). The PLGA-Cy5 NP extravasation through the microleakage is demonstrated. (a) merged; (b) hMNP-FITC; (c) CD31-PE (endothelial marker); (d) PLGA-Cy5 NP. (1) 0 min; (2) 10 min; (3) 35 min; (4) 45 min. Scale bar – 100 μm.

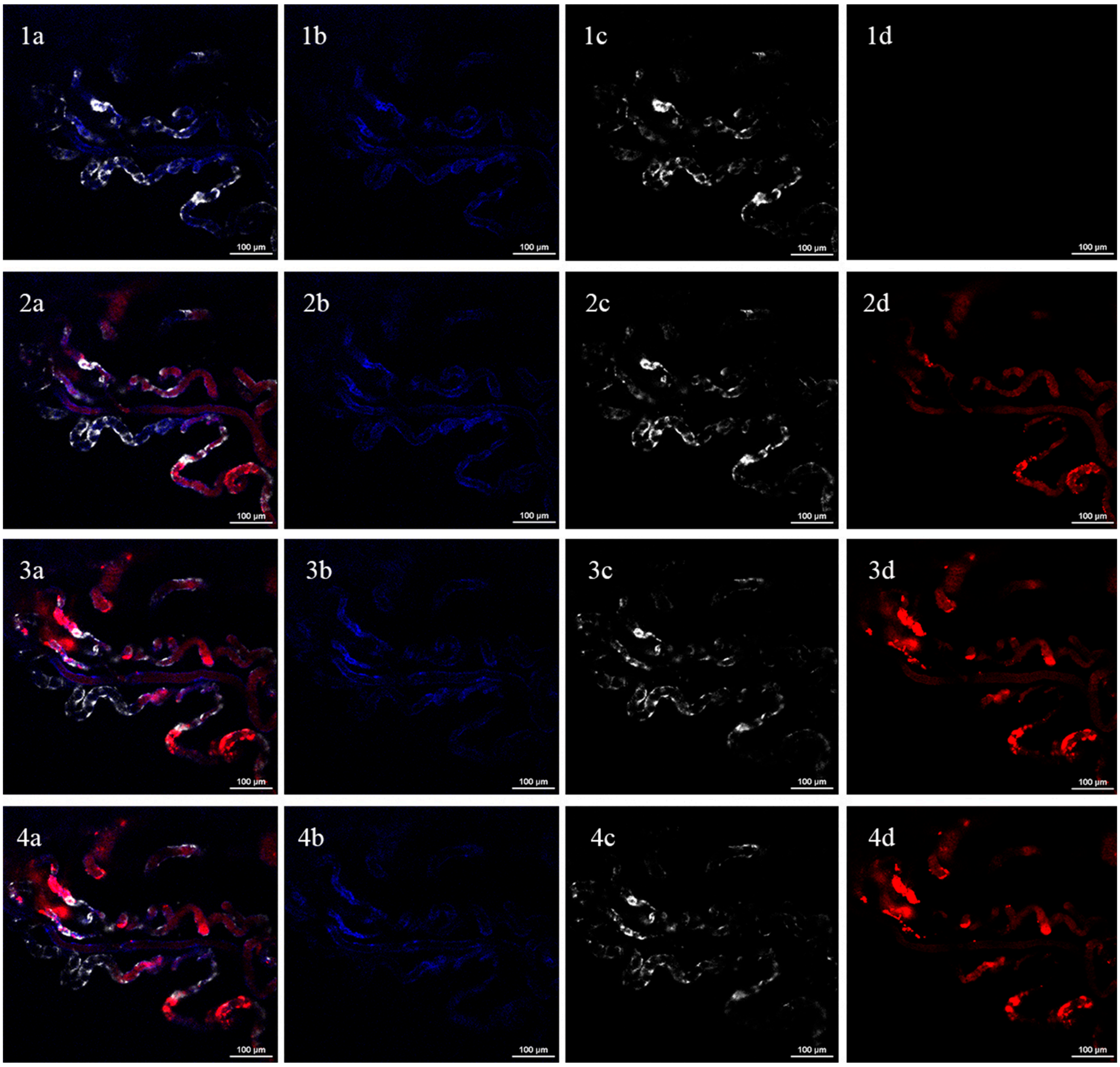

Figure S13. Evaluation of PLGA-Cy5 NP and hMNP-Cy3 distribution in the peritumoral vasculature in 4T1 tumor-bearing mice (5h/1h regimen). (a) merged; (b) CD31-BV; (c) hMNP-Cy3; (d) PLGA-Cy5. (1) 0 min; (2) 10 min; (3) 35 min; (4) 45 min. Scale bar – 100 μm.

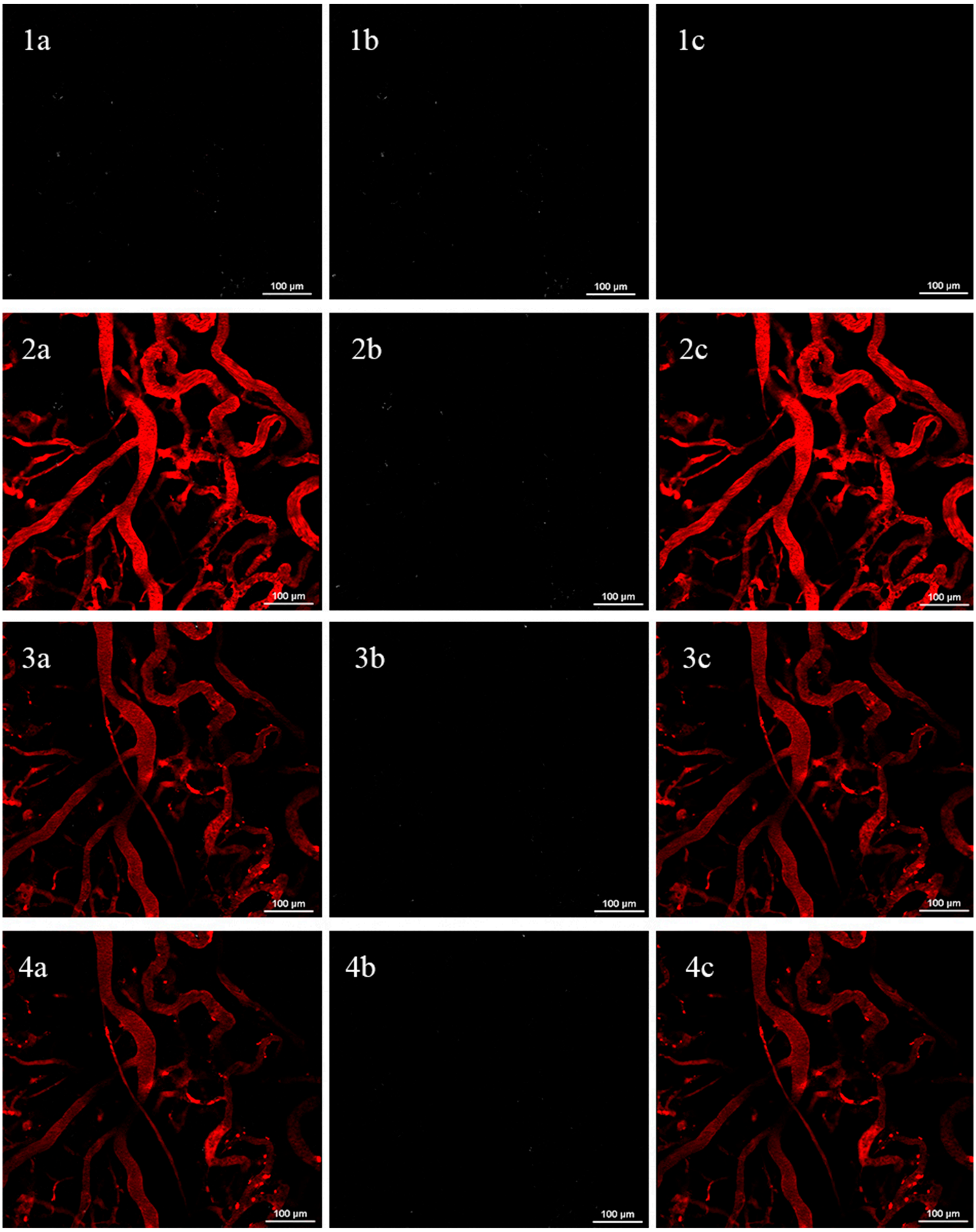

Figure S14. Evaluation of PLGA-Cy5 NP and hMNP-Cy3 distribution in the peritumoral vasculature in 4T1 tumor-bearing mice (24h/1h regimen). (a) merged; (b) hMNP-Cy3; (c) PLGA-Cy5 NP. (1) 0 min; (2) 10 min; (3) 35 min; (4) 45 min. Scale bar – 100 μm.

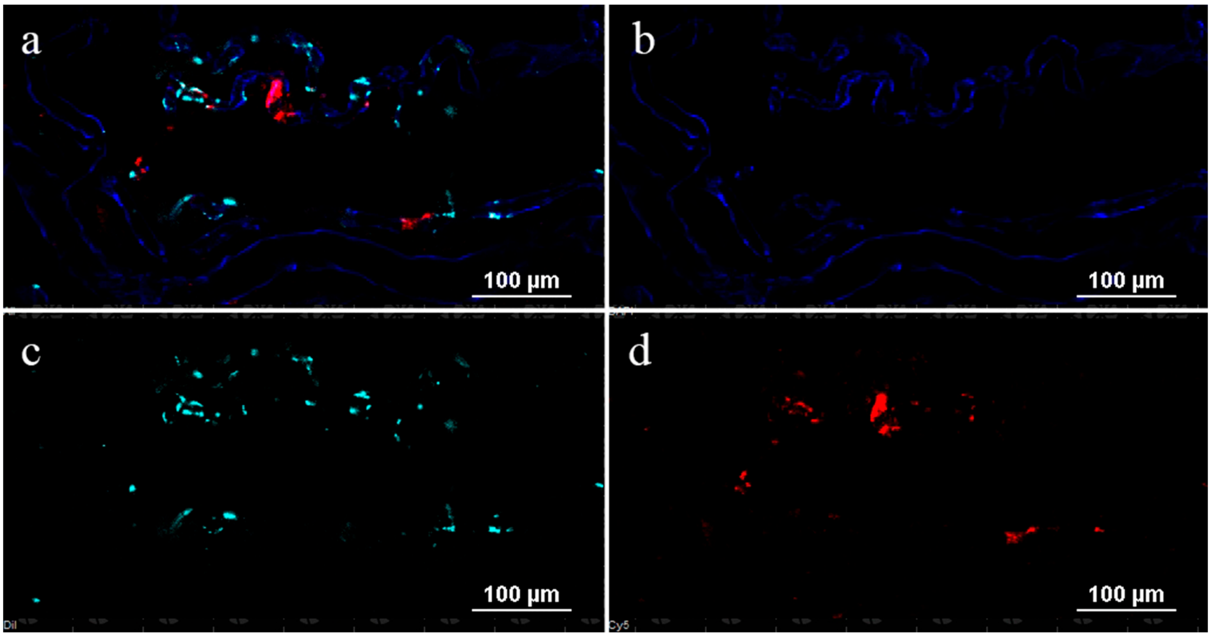

Figure S15. Investigation of PLGA-Cy5 NP and PLGA-Cy3 NP distribution in the peritumoral area in 4T1 tumor-bearing mice (3h/1h regimen). The administration regimen is chosen based on the maximum fluorescence intensity from both NP types. (a) merged; (b) CD-31; (c) hMNP-Cy3; (d) PLGA-Cy5 NP. Scale bar – 100 µm.

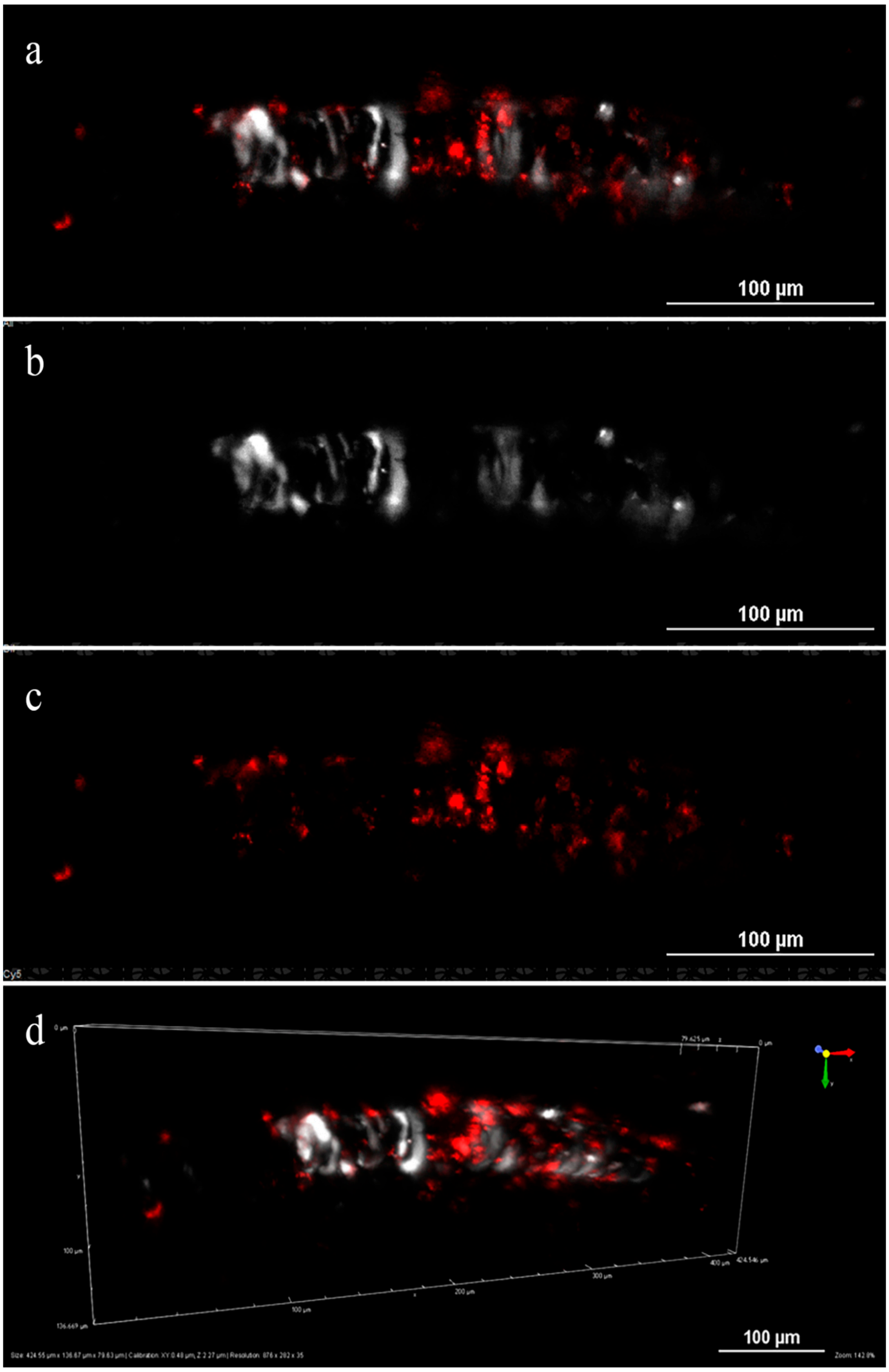

Figure S16. Evaluation of the PLGA-Cy5 NP and hMNP-Cy3 accumulation in the peritumoral vasculature in 4T1 tumor-bearing mice (24h/1h regimen). (a) merged image; (b) hMNP-Cy3; (c) PLGA-Cy5 NP; (d) 3D-reconstruction of the blood circulatory system. Scale bar – 100 µm.

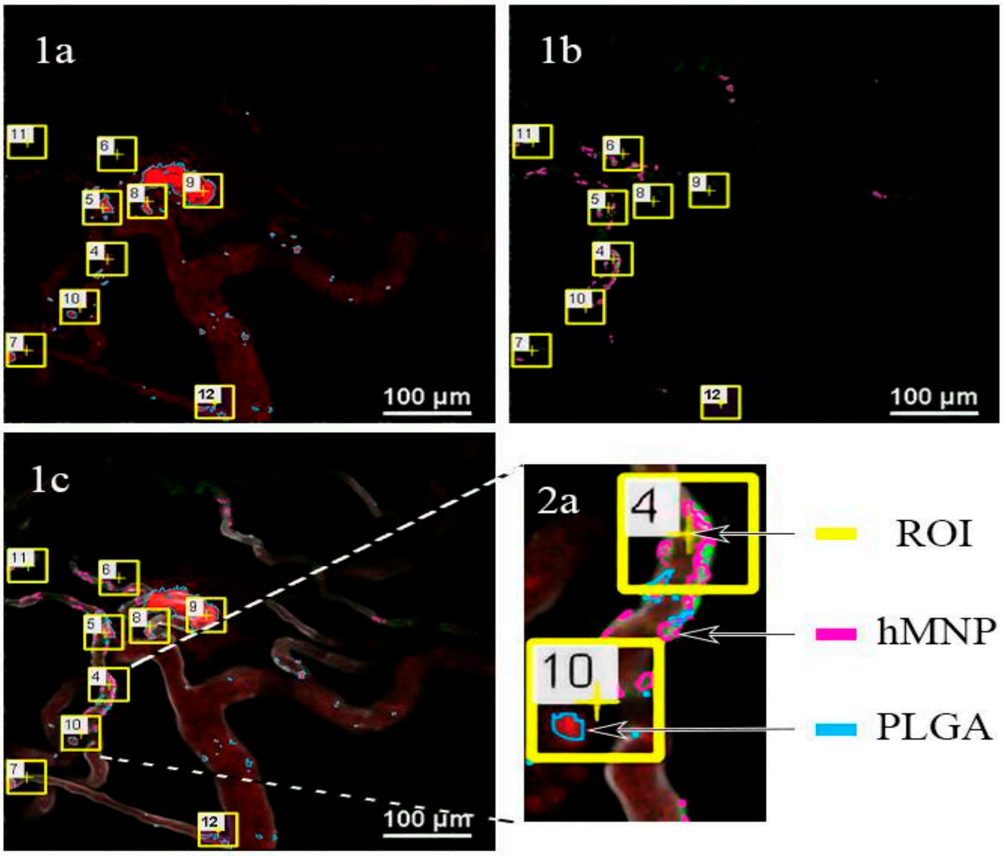

Figure S17. Quantification of hMNP-FITC and PLGA-Cy5 NP accumulation in the tumor and peritumoral area of 4T1tumor-bearing mice. 1a – binary masks (cyan) generated over the regions of PLGA-Cy5 NP accumulation (red channel); 1b – binary masks (magenta) generated over the regions of hMNP-FITC accumulation (green channel); 1c – random generation of ROIs for the frame. Scale bar – 100 µm. 2a – magnification of the ROIs.
